# Infectious Bronchitis Virus in Egypt: Genetic Diversity and Vaccination Strategies

**DOI:** 10.3390/vetsci7040204

**Published:** 2020-12-17

**Authors:** Hassanein H. Abozeid, Mahmoud M. Naguib

**Affiliations:** 1 Department of Poultry Diseases, Faculty of Veterinary Medicine, Cairo University, Giza 12211, Egypt; h.abozeid@cu.edu.eg; 2 Reference Laboratory for Veterinary Quality Control on Poultry Production, Animal Health Research Institute, Agriculture Research Center, Giza 12618, Egypt; 3 Zoonosis Science Center, Department of Medical Biochemistry and Microbiology, Uppsala University, 751 21 Uppsala, Sweden

**Keywords:** chicken, infectious bronchitis virus, genetic evolution, vaccine, disease control, review

## Abstract

Infectious bronchitis virus (IBV) is a highly evolving avian pathogen that has increasingly imposed a negative impact on poultry industry worldwide. In the last 20 years, IBV has been continuously circulating among chicken flocks in Egypt causing huge economic losses to poultry production. Multiple IBV genotypes, namely, GI-1, GI-13, GI-16, and GI-23 have been reported in Egypt possessing different genetic and pathogenic features. Different vaccine programs are being used to control the spread of the disease in Egypt. However, the virus continues to spread and evolve where multiple IBV variants and several recombination evidence have been described. In this review, we highlight the current knowledge concerning IBV circulation, genesis, and vaccination strategies in Egypt. In addition, we analyze representative Egyptian IBV strains from an evolutionary perspective based on available data of their S1 gene. We also provide insight into the importance of surveillance programs and share our perspectives for better control of IBV circulating in Egypt.

## 1. Introduction

Avian coronavirus, or the infectious bronchitis virus (IBV), is responsible for an acute highly contagious viral disease (infectious bronchitis; IB) that tremendously affects the poultry industry worldwide [1]. IBV has a wide host range among avian species. Domestic chickens and pheasants are natural hosts for IBV [2]; however, several IBV strains have been isolated from other domestic avian species and wild birds [2,3].

IBV infects chickens of all ages and induces three clinical forms, namely, respiratory, renal, and reproductive [1]. The morbidity of IBV is quite high, and the mortality depends on strain pathogenicity, the age of the bird, and other factors such as previous vaccination and co-infection with other bacterial or viral pathogens [1]. Clinical symptoms include sneeze, depression, nasal discharge, and tracheal rales, in addition to loss in production and inferior egg quality in layer flocks [2]. IBV can be transmitted through direct contact with infected birds or indirectly through contaminated water or utensils [2].

IBV, a Gammacoronavirus of the family *Coronaviridae*, is 120 nm in diameter with crown-like spikes of 20 nm length. IBV possesses a single-stranded, positive-sense RNA of about 27.6 kb in length comprising 13 open reading frames (ORFs) in the order 5′-UTR-1a-1b-S-3a-3b-E-M-4b-4c-5a-5b-N-6b-UTR-Poly(A)tail-3′ [4]. IBV was reported to be the first coronavirus isolated from chickens in the 1930s in the USA [5]. The first IBV variants were reported in 1950s in the USA [6]. Thereafter, different IBV variants were recorded in a variety of wild bird species [6,7].

Nucleotide heterogeneity is most prevalent in the S1 portion of the S gene and largely contained within three different hypervariable regions (HVRs) corresponding to amino acids 38–67, 91–141 and 274–387 (HVR1, HVR2 and HVR3, respectively) [8].

Phylogenetic classification of IBVs has been globally harmonized based on the sequence analysis of the full-length S1 gene to include 6 genotypes comprising 32 lineages [7]. Genotype I includes 27 distinct viral lineages (temporally ordered GI-1 to GI-27), while each of the remaining genotypes includes one viral lineage (GII-1, GIII-1 GIV-1, GV-1 and GVI-1). Some lineages are widely distributed, especially those used for vaccination such as GI-1 (Massachusetts, H120 and Connecticut) and GI-13 (793/B). Furthermore, GI-19 (QXIBV) and GI-16 (Q1) have also been also reported in diverse localities, including Asia [9,10] Europe [11,12,13], Africa [14], and the Middle East [15,16]. On the other hand, some lineages have been found to be geographically confined to certain countries such as Middle East (GI-23), Asia (GI-7, -15, -18, -22, -24 and GVI-1), North America (GI-8, -9, -17, -20, -25, -27 and GIV-1), South America (GI-11), Europe (GI-21 and GII-1), Africa (GI-26), Australia and New Zealand (GI-5, -6, -10, GIII-1 and GV-1) [7].

## 2. Multiple IBV Genotypes Circulating in Egypt

In Egypt, IBV was first reported in 1954 based on viral isolation and serological identification [17]. The virus was isolated from chickens showing respiratory signs by inoculation in embryonated chicken eggs and further evaluated for pathogenicity in 4-week-old susceptible chicks [17]. From then until 2000, serological investigations using agar gel precipitation test (AGPT), virus neutralization (VN) or hemagglutination inhibition (HI) assays in addition to viral isolation and further identification by such serological tests were the commonly used methods for recognizing IBV infection in chicken farms. During that period, various IBV strains serologically related to the classic strain M41 and the Dutch variant strains D274 and D1466 were recognized [18,19]. Thereafter, reverse transcription polymerase chain reaction (RT-PCR) followed by partial or full S1 gene sequencing has been introduced as a tool for detection and molecular characterization of IBV strains in Egypt [20]. IBV strains closely related to the Dutch variants D274 and D3898 were later isolated and confirmed using partial S1 gene sequencing showing 99.4% and 97.8% nucleotide similarity, respectively [21]. According to the available literature, no other reports about the Dutch variants have been provided since that time.

In 1998, an Egyptian IBV strain were isolated from 38-day-old broiler chickens suffering from respiratory and renal symptoms in the Beni-Suef governorate, and was later characterized based on the HVR3 sequence of the S1 gene in 2001. The isolated strain (Egypt/Beni-Suef/01; GenBank accession number AF395531) was identified as the Egyptian variant-1 strain found to be unique to Egypt, but closely related to the nephropathogenic strains IS/720/99 (GenBank accession number AY091552) isolated in Israel, showing 97.6% sequence similarity, with six amino acid and eight nucleotide substitutions [22]. The Egyptian variant-1 strain also shared 96.6% amino acid identity with the nephrogenic Israeli variant strain IS/885/00 (GenBank accession number AY279533) [23].

**Figure 1:**
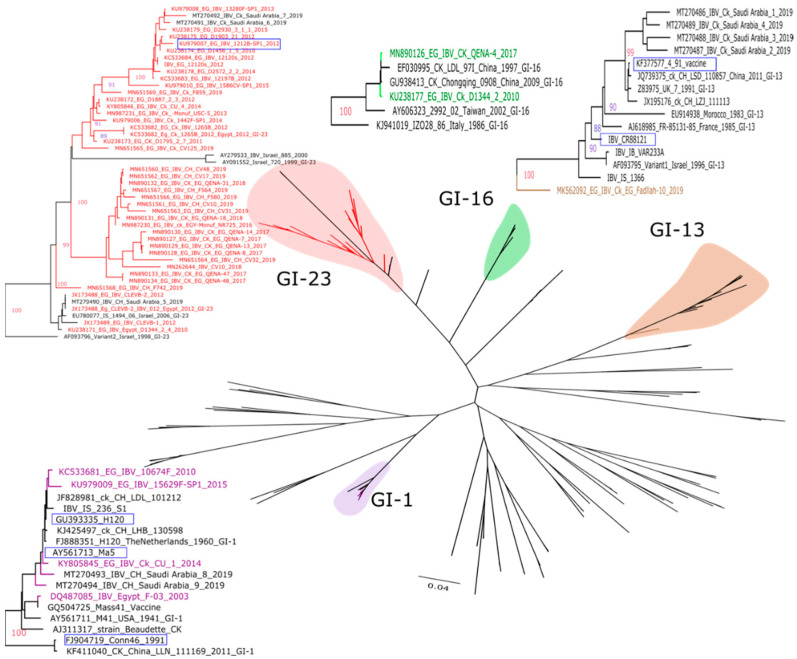
Phylogenetic tree of the nucleotide sequences of full S1 gene of Egyptian IBVs. Phylogenetic analysis was based on genotype classification of the S1 gene [7]. Maximum likelihood calculations were done using the IQTree software under the best fit model (TIM3 + F + I + G4) according to the Bayesian criterion. Egyptian IBVs were colored based on the genotype (purple = GI-1, brown = GI-13, green = GI-16, and red = GI-23). Vaccine strains used in Egypt are shown in the blue box. The strain names, locations, and accession numbers of different lineages are fully described in Valastro et al. 2016 [7].

In 2003, a nephropathogenic Egyptian IBV strain, named Egypt/F/03 (GenBank accession number DQ487085), was isolated from broiler chickens suffering from severe renal and respiratory symptoms [24]. The isolated strain showed 97% nucleotide similarity of the full S1 gene sequence with the H120 vaccine strain belonging to classic GI-1 lineage (Figure 1). However, a protection study showed low protection using the H120 vaccine against the Egyptian strain Egypt/F/03. Subsequently, several studies reported the isolation of classic GI-1 lineage strains from vaccinated flocks indicating the protection inability of classic vaccines against the Egyptian classic IBV strains [25,26,27], suggesting that few amino acid changes could result is generating an escape mutant virus.

Later, in 2011, two IBV variant strains, namely, Ck/Eg/BSU-2/2011 and Ck/Eg/BSU-3/2011, were identified, and their HVR3 sequence analysis revealed distinction from any known Egyptian variants or vaccine serotypes and designated as Egyptian variant-2 [28]. The Egyptian variant-2 strains Ck/Eg/BSU-2/2011, and Ck/Eg/BSU-3/2011 showed nucleotide similarity of 89% and 87% with the Egyptian variant-1 strain Egypt/Beni-Suef/01, 91%, and 88% with the Israeli variant strain IS/885/00, and 89% and 88% with Israeli IS/1494/06, respectively. No complete S1 gene sequence is available for these parental Egyptian variant strains. However, many Egyptian IBV strains that are closely related to those parental Egyptian variants have been isolated and full S1 gene sequencing was carried out by many researchers (Figure 1) [20,26,29,30].

These two Egyptian variant subgroups (Egyptian variant-1 and Egyptian variant-2), later identified as GI-23 lineage, represent the most prevalent lineage in Egypt, which has been circulating in all kinds of chicken flocks until now [30,31,32,33]. Recently, Egyptian variant-1 and Egyptian variant-2 were isolated from different wild bird species (house sparrow, teal, cattle egret, and quail) at the upper part of Egypt indicating spillover transmission from domestic poultry to wild birds [3]. This could interpret the recent detection of the GI-23 lineage strains in Europe [34] that had previously been identified as an indigenous Middle Eastern lineage for nearly 20 years [7].

In 2012, the novel variant IBV strain VSVRI_F3 (GenBank accession number KP729419) was isolated from boiler flock suffering from renal signs in Fayoum governorate, and partial S1 gene sequence analysis showed 99.1% nucleotide identity to the Q1 strain, later identified as GI-16 lineage [32]. Similarly, in 2017, the strain IBV/CK/EG/QENA-4/2017 (GenBank accession number MN890126) was isolated from unvaccinated broiler flock in upper Egypt (Qena), and full S1 gene sequence analysis showed close relatedness to GI-16 lineage strains previously isolated in China, Italy and Vietnam and with high nucleotide (99.7–99.9%) and amino acid (99.2–99.8%) identities (Figure 1) [31]. Historically, the Q1 strain was first isolated in China between 1996 and 1998 [35], and then Q1-related strains were reported in many countries including Middle Eastern countries like Iraq, Jordan, and Saudi Arabia [15].

In 2019, IBV/CK/EG/Fadllah-10/2019 (GenBank accession number MK562092), a pathogenic 4/91 IBV strain belonging to GI-13 lineage IBV was isolated from Ma5-vaccinated layer flock and identified based on full S1 glycoprotein analysis. The isolated strain showed 88–90% amino acid identity with the currently used 793/B vaccine group (CR88 and 4/91) and was described as a new genotype-lineage expression (GI-13 lineage) in Egypt (Figure 1) [20].

The phylogenetic analysis conducted within this review, based on full S1 sequencing of all available Egyptian IBVs (n = 48), revealed that Egyptian IBVs are clustered within four different lineages of genotype I (GI-1, GI-13, GI-16, and GI-23) (Figure 1). Phylogenetic analyses were constructed using the maximum likelihood methodology based on Bayesian criterion after selection of the best-fit models by using IQ-TREE software version 1.1.3 [36].

## 3. Genetic Drift and Recombination of the Egyptian IBV Strains

The high diversity of the IBV strains is attributed mainly to the mutations and/or genetic recombination that frequently occur in coronaviruses [7,37]. The high nucleotide error rate of viral RNA polymerase (10^−3^–10^−4^) is the main reason for the high mutation rate of IBV [38]. Mutations (insertion, deletions, and substitutions) in few immunodominant amino acids—especially in the S1 subunit—could result in emergence of a new serotype which escapes the preexisting immunity [39,40,41]. When two different strains infect the same cell, genetic recombination is very likely to happen because of the template-switching mechanism of the viral RNA polymerase [42,43], leading to creation of a new variant that is quite distinct from the parental strains.

In Egypt, evidence for genetic drift were reported to be involved in the evolution of Egyptian IBV strains. Genetic drift was first described in the classic strain Egypt/F/03, whose full S1 gene sequence analysis showed 97% nucleotide and amino acid identity to H120 strain, with 14 amino acid substitutions, including—but not limited to—HVR1, 2 and 3 [24]. The location of amino acid substitution, namely S130F and L141F, has been previously suggested to be responsible for altered pathogenicity [44], explaining the high virulence of the emerged Egyptian strain Egypt/F/03 and the protection inability of the closely related H120 vaccine.

In addition, the HVR3 sequence analysis of the Egyptian variant-1 strains Ck/Eg/BSU-1/2011, Ck/Eg/BSU-4/2011 and Ck/Eg/BSU-5/2011 revealed an amino acid substitution (W243R) compared to the parental strain Egypt/Beni-Suef/01. Moreover, Ck/Eg/BSU-1/2011 showed another amino acid substitution, namely, R355M [28]. In the same study, HVR3 analysis of Egyptian variant-2 strains Ck/Eg/BSU-2/2011 and Ck/Eg/BSU-3/2011 showed seven and eleven amino acid substitutions compared with Egyptian variant-1 strain Egypt/Beni-Suef/01, respectively. Accordingly, the Egyptian variant-2 strains were described as new variant subgroup that are quite distinct from the ancestor variant-1 strain [28].

Based on the amino acid sequence analysis of full S1 glycoprotein, locally isolated IBV strains belonging to the two Egyptian variant subgroups, Egy/var-I and Egy/var-II, were clustered within G1-23 and exhibited genetic drift including a deletion mutation at amino acid position 63 as well as a substitution at residue 169 of the S1 glycoprotein [29]. Amino acid sequence analysis suggested that the Egyptian variant subgroups differ in genetic features from the classical vaccine group, the H120 lineage [29]. Subsequent studies of pathogenicity, in comparison with the classical genotypes, showed that the Egyptian IBV variants can result in up to 50% mortality in chickens [25]. In another study, based on the full genome sequence analysis of the Egyptian IBV classic (GI-1) strain IBV/EG/CU/1/2014 (GenBank accession number KY805845), genetic drift including a novel 15-nt deletion in the 4b-4c overlapping region in addition to multiple point mutations within the genome of the studied strain were reported [26]. This Egyptian IBV strain was described as a revertant from the vaccine strain H120 that could be arisen by acquiring mutations and efficiently adapting in chicken flocks. In a recent study, the newly reported GI-13 Egyptian strain IBV/CK/EG/Fadllah-10/2019 exhibited several distinct point mutations within the three HVRs compared to the commercially used 4/91 vaccine strain [20].

Recombination also plays a major role in the evolution of IBVs [45,46,47]. Both inter- and intra-genotypic recombination were described among the Egyptian IBV strains [20,26,29]. Based on the sequence analysis of the full S1 gene, Zanaty and coauthors [29] reported evidence for intra-genotypic recombination between the Egy/var-I (IBV/EG/CLEVB1/2012; GenBank accession number JX173489) and Egy/Var-II (IBV/EG/12120s; GenBank accession number KC533684) of GI-23 lineage composing the recombinant virus (IBV-EG/1586CV-2015; GenBank accession number KU979010). In another study, based on the sequence analysis of the full genome, the variant IBV strain (IBV/EG/CU/4/2014; GenBank accession number KY805846) showed evidence for at least three inter-genotypic recombination events including three different IBV strains, namely, IT02, 4/91 and H120 [26]. Abozeid et al. suggested that the variant IBV strain could be created by recombination between the circulating variant strains and the vaccine strains (H120 and 4/91) used at that time. Similarly, in 2019, the Egyptian IBV isolate IBV/CK/EG/ Fadllah-10/2019; GenBank accession number MK562092, was described as a result of recombination between the Egyptian variant genotype (GI-23 lineage) and 4/91 genotype (GI-13 lineage) based on the analysis of the full S1 gene sequence [20].

Recently, the Egyptian variant strain EGY/NR725/2016 (GenBank accession number MN987230) revealed intra-genotypic recombination for genes S1, 3ab and E with another Egyptian variant genotype (GI-23 lineage). The same isolate also revealed inter-genotypic recombination for its entire gene 6b with a close match to QX-like genotype (GI-19 lineage) [30]. The recombination of the Egyptian IBV strains with QX-like strain and experience from the field make us suggest the presence of QX-like IBV in Egypt which might be “flying under the radar” of the national surveillance among poultry population in Egypt.

## 4. IBV Vaccines in Egypt

Control of IB relies mainly on the use of live-attenuated and inactivated IBV vaccines, in addition to implementation of good biosecurity. Live-attenuated vaccines are mainly used for immunization of meat-type chickens and for priming of future layer and breeder flocks. Live-attenuated IBV vaccines elicit good cellular and humoral immune responses that usually provide good protection against homologous challenge [48]. However, protection against heterologous challenge is limited [49]. Combinations between two antigenically different IBV strains was suggested to provide brooder spectrum of protection against different variant strains [50]. Inactivated vaccines are commonly used for boosting the layer and breeder flocks to ensure a long-lasting humoral immunity during the period of egg production, and to transfer maternal immunity from the breeders to their progeny [51]. In Egypt, live-attenuated and inactivated IBV vaccines are extensively used to control the disease. Classic vaccine strains (H120 and Ma5) have been widely used as a routine measure to control the disease in Egypt. Since 2012, the variant vaccine strains (D274 and 793B) have been introduced to be implemented within the vaccination regimens to control the IB outbreaks in Egypt. Despite the intensive vaccination, classic and variant IBV strains associated with disease outbreaks have been frequently reported in Egypt [25,26,27,33,52]. Recently, a homologous live-attenuated VAR2 vaccine was produced from the Egyptian variant-2 strain Eg/1212B/2012 (GenBank accession number JQ839287) by a national factory of vaccine production and is commercially licensed for use to combat IB in Egypt [48]. Experimentally, the VAR2 vaccine provided excellent protection against the homologous IBV challenge [48]. In a research study, a Newcastle disease virus-vectored IBV vaccine was developed using the full S gene of the Egyptian variant-2 strain as immunogenic insert [53]. The developed vaccine provided clinical protection against the homologous challenge after single vaccination of 1-day old SPF chicks, and further showed significant reduction of tracheal viral load after prime-boost vaccination at 1 and 14 day of age. However, the co-circulation of multiple IBV serotypes/genotypes along with other respiratory and immunosuppressive viruses, poor biosecurity measures and overcrowding make the field situation overly complicated in Egypt.

## 5. Perspectives and Recommendations for a Better Control Strategy

To conclude, four genotype lineages (GI-1, GI-23, GI-16 and GI-13) are reported to be co-circulating in chicken farms in Egypt. GI-1 includes the classic wild strains in addition to the vaccine-like strains that are probably emerged by acquiring mutations through multiple passages in chicken flocks. GI-23 includes the two Egyptian variant subgroups (Egy/Var-1 and Egy/Var-2) that are undergoing continuous evolution by genetic drift and genetic recombination either within the same genotype (intra-genotypic) or with different genotypes (inter-genotypic). GI-16 includes the newly introduced Q1-like strains. It is still unclear if this lineage is introduced from China, Europe or from the neighboring Middle Eastern countries. Much is yet unknown about the evolution of this genotype. GI-13 includes the 4/91-like strains which is suggested to be emerged from the currently used 4/91 vaccine strain. Therefore, updated epidemiological and genetic features of IBV in Egypt should be accurately determined. Due to the recombinant nature and genetic diversity of the IBV, phylogenetic grouping based on a specific fragment of a gene might change when investigating another fragment of the same gene, the entire gene, or the whole genome. Similarly, Valastro and coauthors found that phylogenetic analysis based on HVRs1 and 2, and HVR3 revealed inconsistent classification outcomes and are inequivalent to those based on the full-S1 gene analysis [7]. Hence, molecular analysis of at least the full S1 gene of geographically representative IBV strains is recommended on regular basis to obtain meaningful results. However, to provide a comprehensive overview about the evolution of IBV, full genome analysis is rather recommended to investigate the recombination hot spots other than S1 gene, which could have a great influence on the viral behavior in terms of replication, pathogenicity and immunogenicity as well [45,54]. In addition, the role of wild birds in transmission of IBV strains from Egypt to other countries and vice versa should be investigated.

The abuse of the multiple IBV vaccine strains plays a key role in the evolution of the Egyptian strains; by reverting to virulence and/or by genetic recombination with the circulating field strains. Since the fast evolution rate of IBV strains is partly attributed to the live-attenuated IBV vaccines [55], the need for a novel vaccine that could be safely used to protect chickens without increasing the potentiality of creating new variants is rising. Until such vaccine is developed, the current vaccination strategy should be wisely planned to use the most relevant protecteotype strain based on in vivo protection studies rather than genotype clustering because as few as 2–3% mismatching in certain immunodominant amino acids between two IBV strains could significantly reduce the cross-protection [40,41]. Moreover, the vaccination procedures and precautions must be strictly followed to ensure a full dose of viable vaccine uptake by the host. In the meantime, the health of the birds’ immune system should be maintained by avoiding all immunosuppression factors such as mycotoxins and managemental errors like poor ventilation, overcrowdings, and failure to adjust the temperature and humidity suitable for each stage of production, leading to vaccination failure and make the herd vulnerable to infection. Biosecurity comes on the top of that to prevent exposure to infection as much as possible.
